# Detection of disseminated tumor cells in bone marrow predict late recurrences in operable breast cancer patients

**DOI:** 10.1186/s12885-019-6268-y

**Published:** 2019-11-21

**Authors:** Kjersti Tjensvoll, Oddmund Nordgård, Maren Skjæveland, Satu Oltedal, Emiel A. M. Janssen, Bjørnar Gilje

**Affiliations:** 1 Department of Haematology and Oncology, Stavanger University Hospital, N-4011 Stavanger, Norway; 2 Laboratory for Molecular Biology, Stavanger University Hospital, N-4011 Stavanger, Norway; 3 Department of Pathology, Stavanger University Hospital, N-4011 Stavanger, Norway

**Keywords:** Disseminated tumor cell, DTC, Dormancy, Breast cancer, Proliferation, Mitotic activity index, Late recurrence

## Abstract

**Background:**

Operable breast cancer patients may experience late recurrences because of reactivation of dormant tumor cells within the bone marrow (BM). Identification of patients who would benefit from extended therapy is therefore needed.

**Methods:**

BM samples obtained pre- and post-surgery were previously analysed for presence of disseminated tumor cells (DTC) by a multimarker mRNA quantitative reverse-transcription PCR assay. Updated survival analyses were performed on all patient data (*n* = 191) and in a subgroup of patients alive and recurrence-free after 5 years (*n* = 156). DTC data were compared to the mitotic activity index (MAI) of the primary tumors. Median follow-up time was 15.3 years.

**Results:**

Among the 191 patients, 49 (25.65%) experienced systemic relapse, 24 (49%) within 5–18 years after surgery. MAI and pre- and post-operative DTC status had significant prognostic value based on Kaplan–Meier analyses and multiple Cox regression in the overall patient cohort. With exclusion of patients who relapsed or died within 5 years from surgery, only pre-operative DTC detection was an independent prognostic marker of late recurrences. High MAI (≥10) did not predict late recurrences or disease-specific mortality.

**Conclusion:**

Pre-operative DTC detection, but not MAI status, predicts late recurrences in operable breast cancer.

## Background

Breast cancer patients are at risk of developing disease relapse decades after curative treatment because of the presence of minimal residual disease [1]. Minimal residual disease is caused by spread of invasive tumor cells from the primary tumor through the circulation to distant sites [2, 3]. Once in circulation, interaction with platelets seems to contribute to circulating tumor cell (CTC) survival as well as enhanced extravasation, especially to the bone microenvironment [4–6]. In breast cancer, tumor cells often migrate to the bone marrow (BM) where these disseminated tumor cells (DTCs) can survive for years by entering a dormant state. This is a prolonged quiescent state, in which tumor cells are present, but disease progression is not clinically manifested. Two mechanisms are believed to maintain tumor cell dormancy: single-cell dormancy and/or micrometastatic dormancy [7]. Single-cell dormancy, or cellular dormancy, is characterised by a state in which the tumor cells are non-proliferative and thus assumed to be resistant to traditional chemotherapeutics targeting proliferating cells [7]. In contrast, the micrometastatic dormancy model, which is supposed to be linked to more aggressive breast cancer, involves slowly proliferating tumor cells that are counterbalanced by cell death from impaired vascularisation or immunesurveillance, which prevents tumor growth [7]. However, dormant DTC survival in the BM depends on pro-survival signals from the microenvironment, as well as on development of complex immune evasion mechanisms in which interference with major histocompatibility complex–mediated antigen presentation seems to be important [8–11]. Breast cancer recurrence after a long asymptomatic period, even more than 20 years after the initial diagnosis, is believed to arise from an interruption of this dormant DTC state, possibly initiated by microenvironmental factors in the colonised tissue [12]. Thus, accurate identification of breast cancer patients at risk for late recurrences is needed to identify candidates for extended therapy and improve survival. Unfortunately, few studies report sufficiently long follow-up of breast cancer patients.

The prognostic relevance of DTC detection has been demonstrated by us and others [13–18], but only one other study has investigated the clinical significance of DTC detection for prediction of late recurrences by including long-term follow-up data [19]. For this reason, we evaluated the capacity of DTC detection prior to and after surgery to predict long-term outcome in 191 operable, prospectively recruited breast cancer patients in comparison to other well-established prognostic markers such as mitotic activity index (MAI) and lymph node status in a retrospective analysis [14–16]. To our knowledge, this study involves the longest follow-up reported in breast cancer studies investigating the clinical significance of DTC detection.

## Methods

### Patient cohort

The patients (*n* = 191) included in this study were consecutively recruited during the years 1998–2000. All patients had non-metastatic (M0) breast cancer, and the median age was 56 years (range 25–86 years). BM samples (20 ml in heparin) were drawn unilaterally from the posterior iliac crest under general anaesthesia immediately prior to surgery (BM1, *n* = 191) as well as 3 weeks (BM2) and/or 6 months (BM3) after surgery (*n* = 154) [16]. In addition, BM aspirates were obtained from 26 healthy women included as a control group. Written informed consent was obtained from all participants, and the project was approved by the Regional Committee for Medical and Health Research Ethics.

With regard to the treatment, all patients were treated according to the national guidelines in Norway at this time as previously described in Farmen et al., 2008 [20]. In brief, the patients either underwent a modified radical mastectomy or lumpectomy with breast-conserving surgery. Patients operated by lumpectomy received additional treatment in terms of radiotherapy to the breast. Level I or II axillary lymph node (LN) dissection was performed on all patients. Adjuvant combination therapy, including either CMF (cyclophosphamide, metotrexate and 5-fluorouracil) or FEC (5-fluorouracil, epirubicin and cyclophosphamide), was given to all high risk patients. In addition, 20 mg tamoxifen was given daily for 5 years to high-risk patients having a positive or uncertain hormone receptor status [20].

Patient follow-up data were collected from medical records at the hospital and from the patients` primary physicians. Information on time of death was obtained from the hospital records, which is updated based on information from the National Registry in Norway. The last follow-up was registered in August 2016, and the median follow-up time was 15.3 years (range 0.06–18.15 years). Early recurrence was defined as relapse within 5 years, and late recurrence was defined as relapse occurring more than 5 years after the initial surgery.

### mRNA marker analyses in BM samples

BM lysates were prepared from buffy coat, and total RNA was isolated and transcribed to cDNA followed by amplification of a multimarker panel consisting of keratin 19 (KRT19), mammaglobin (hMAM) and TWIST1 mRNA in a LightCycler 480 (Roche Applied Science) instrument as previously described [14–16, 20]. After data analysis, a BM sample was considered to have presence of DTCs when at least one of the mRNA markers (i.e., KRT19, hMAM or TWIST1) exceeded the highest mRNA level in BM samples from the control group (*n* = 26), which was used as a threshold for normal expression [15, 16].

### Assessment of MAI in primary tumors

The assessment of MAI was performed by a trained technician at × 400 magnification by systematic counting of well-defined mitoses in haematoxylin and eosin–stained slides of the primary tumor according to the Multicenter Mammary Carcinoma Project protocol [21–23], and as previously described in Gilje et al. [24]. The resulting total number of mitoses in the 10 fields of vision was defined as the MAI, and dichotomised as being < 10 or ≥ 10 where low MAI (< 10) indicates a favourable prognosis and a high MAI (≥10) a worse prognosis [25].

### Statistical analyses

All statistical analyses were performed in SPSS version 24.0 (www.spss.com) and R version 3.3.3, with a two-sided *p* value ≤0.05 considered as statistically significant. Multiple testing were not corrected for, and missing data were excluded from all analyses. Fisher’s exact test were used to test for any relations between the multimarker BM expression and various endpoints. Kaplan–Meier estimates of clinical outcome were determined from primary surgery to A) systemic disease recurrence (systemic recurrence-free survival); B) death related to progression of breast cancer (breast cancer–specific survival); and C) death from any cause (overall survival). In this respect, pre-operative DTC status refers to DTC detection in BM samples obtained prior to surgery (BM1), while post-operative DTC status refers to DTC detection in BM samples obtained 3 weeks (BM2) and/or 6 months (BM3) after surgery. In addition, long-term survival was analysed separately by including only patients who were recurrence-free and alive 5 years (*n* = 156) after surgery and subtracting 5 years from their survival times.

Univariable Cox regression was performed to evaluate whether pre-operative DTC status, post-operative DTC status, pre- and post-operative DTC status, LN status, tumor size, tumor grade, age, oestrogen receptor (ER) status, progesterone receptor (PR) status, adjuvant therapy and MAI status were associated with systemic recurrence-free- and breast cancer–specific survival. Multivariable Cox regression was also performed to evaluate which of the listed risk factors independently could predict reduced systemic recurrence-free survival and breast cancer–specific survival. Pre- and post-operative DTC status could be entered into the same model, because they were not significantly associated. The multivariable analyses were performed using both forward and backward selection of covariates into the Wald model. However, only data from the backward selection is presented. A 95% confidence interval (CI) and a maximum of 50 iterations were used in these analyses.

## Results

### DTC detection and proliferation in operable breast cancer patients with long-term follow-up

In this study, 49/191 (26%) early-stage breast cancer patients experienced systemic relapse during a median follow-up of 15.3 years (range 0.1–18.2 years). Of these, 25 (51%) patients relapsed within 0–5 years after primary tumor resection, 9 (18%) patients relapsed between 5 and 10 years, and 15 (31%) relapsed after more than 10 years from their breast cancer surgery. Furthermore, 38/191 (19.9%) patients died from breast cancer during follow-up, compared to 37 (19.4%) patients who died from causes other than breast cancer.

Regarding BM assessments, 30/191 (15.7%) patients were DTC-positive in pre-operative BM samples, while 23/154 (14.9%) patients (missing data from 37 patients) were DTC-positive in post-operative BM samples. Of interest, 16/30 (53.3%) pre-operative DTC-positive patients relapsed compared to 33/161 (20.5%) DTC-negative patients (*p* < 0.001). Moreover, among the 16 relapsed pre-operative DTC-positive patients, 9 (30%) relapsed within 5 years while 7 (23.3%) relapsed within 5–18 years after their breast cancer surgery. When looking at death reports, 17 (56.7%) of the 30 pre-operative DTC-positive patients died from their disease compared to 58 (36%) of 161 DTC-negative patients (*p* = 0.042). In contrast, nine pre-operative DTC-positive patients had no reported disease relapse after > 15 years of follow-up. Of the 23 post-operative DTC-positive patients, 11 (47.8%) relapsed compared to 29/131 (22.1%) DTC-negative patients (*p* = 0.018). Four (36.4%) post-operative DTC-positive patients relapsed 5–18 years after surgery, and three of them died of breast cancer. Comparison of clinicopathological parameters and DTC status in BM before primary surgery is shown in Table 1.

**Table 1:** Comparison of the clinicopathological parameters of the operable breast cancer patients (*n* = 191) according to DTC status in bone marrow before primary surgery

Variable	No. of patients(*n* = 191)	Pre-operative DTC status		*P*-values
		Positive (*n* = 30)	Negative (*n* = 161)	
Age (%)				**0.028**
< =55 years	94	9 (10)	85 (90)	
> 55 years	97	21 (22)	76 (78)	
Lymph node status (%)				**0.016**
pN0	133	15 (11)	118 (89)	
pN1–2	58	15 (26)	43 (74)	
Tumor size (%)				0.816
pT1	145	22 (15)	123 (85)	
pT2–4	46	8 (17)	38 (83)	
Tumor grade (%)				0.421
1	67	8 (12)	59 (88)	
2	75	15 (20)	60 (80)	
3	46	7 (15)	39 (85)	
Unknown	3			
Estrogen receptor status (%)				0.612
ER positive	152	23 (15)	129 (85)	
ER negative	36	7 (19)	29 (81)	
Unknown	3			
Progesterone receptor status (%)				0.549
PgR positive	91	13 (14)	78 (86)	
PgR negative	91	17 (19)	74 (81)	
Unknown	9			
Histological type (%)				0.088
Ductal	151	20 (13)	131 (87)	
Lobular	22	7 (32)	15 (68)	
Other	18	3 (17)	15 (83)	
MAI status (%)				0.678
High MAI (≥10)	60	11 (18)	49 (82)	
Low MAI (< 10)	119	19 (16)	100 (84)	
Adjuvant therapy (%)				**0.029**
Chemotherapy	21	4 (19)	17 (81)	
Endocrine therapy	25	9 (36)	16 (64)	
Chemo- and endocrine therapy	32	3 (9)	29 (91)	
No therapy	113	14 (12)	99 (88)	

*p*-value ≤0.05 are in bold

Counting of mitoses in the primary tumors for assessment of the MAI score was possible only for 179 of the 191 included patients. Among these, 60 had high MAI (≥10), indicating high proliferation in the primary tumor. Of these patients, 21 (35%) relapsed, 19 of them within the first 5 years from the diagnosis, and 20 (33%) patients died because of breast cancer.

### Survival analysis

**Fig. 1:**
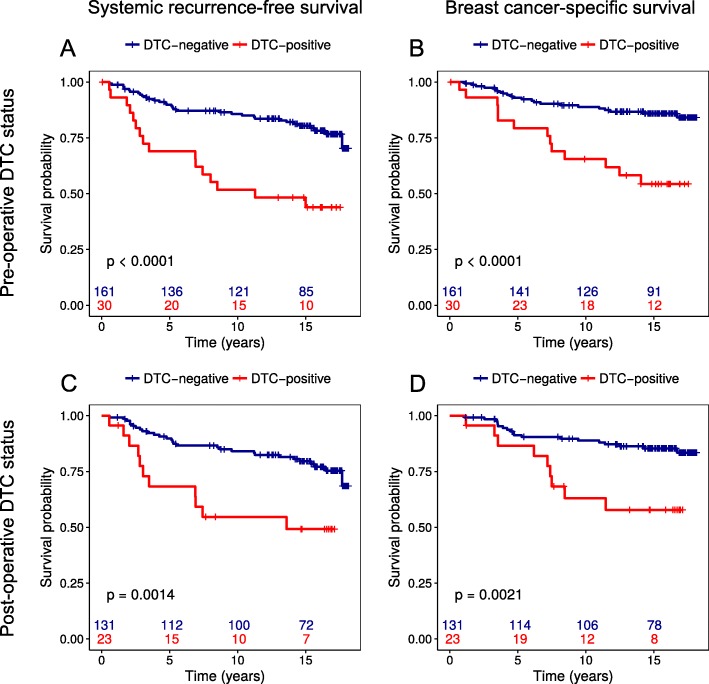
Survival analyses according to presence of disseminated tumor cells (DTCs) in bone marrow (BM) samples obtained prior to surgery (**a** and **b**) and 3 weeks and/or 6 months after surgery (**c** and **d**) from 191 operable breast cancer patients with a median follow-up of 15.3 years

Kaplan–Meier analyses revealed that patients with DTCs present in their BM prior to surgery had significantly shorter systemic recurrence-free- (*p* < 0.001) and breast cancer–specific survival (*p* < 0.001) compared to DTC-negative patients (Fig. 1a and b). This pattern was also seen for overall survival (*p* = 0.013; Additional file 1: Figure S1-A). In addition, stratification of the data for adjuvant treatment demonstrated a significantly reduced systemic recurrence-free survival among the DTC-positive patients receiving adjuvant treatment (*p* = 0.001).

**Fig. 2:**
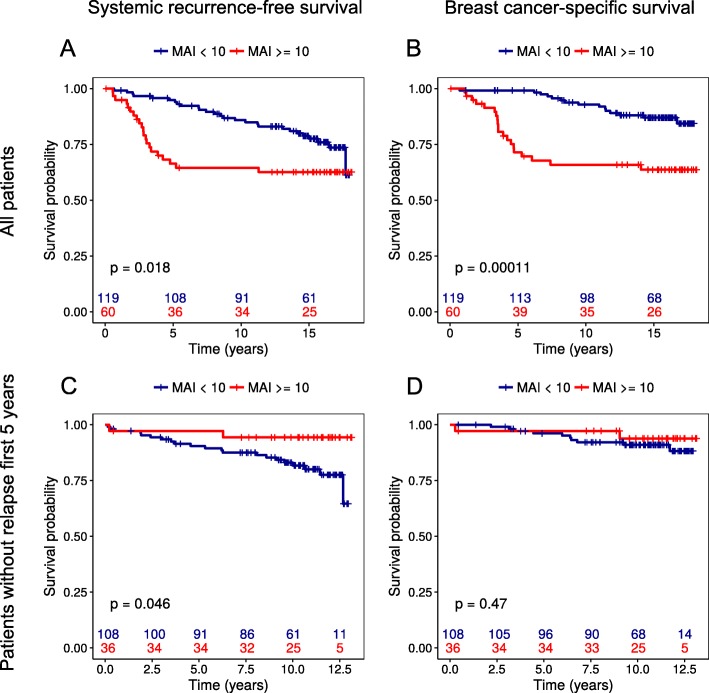
Kaplan–Meier survival estimates according to high (MAI ≥ 10) or low (MAI < 10) proliferation measured by the mitotic activity index (MAI) in tissue from operable breast cancer patients. **a** and **b**) MAI status in all patients (*n* = 191) with median 15.3 years of follow-up. **c** and **d**) MAI status in patients (*n* = 144) who did not experience disease recurrence or died during the first 5 years after surgery. In this case, the first 5 years were subtracted from all survival times prior to analysis

Investigations of the prognostic relevance of persistent DTCs in BM, by analyses of BM samples obtained 3 weeks and/or 6 months after primary surgery demonstrated that DTC status after surgery was a prognostic marker for both systemic recurrence-free- (*p* = 0.001) and breast cancer–specific survival (*p* = 0.002) after a median 15.3 years of follow-up (Fig. 1c and d). Furthermore, persistent DTCs in BM were associated with significantly reduced overall survival (*p* = 0.001; Additional file 1: Figure S1-B). Regarding primary tumor MAI status, a high MAI count was also a significant predictor of shorter systemic recurrence-free-survival (*p* = 0.018; Fig. 2a), breast cancer–specific survival (*p* < 0.001; Fig. 2b) and overall survival (*p*=0.026; Additional file 2: Figure S2-A). These survival curves show, however, that most disease recurrences and breast cancer–related deaths in patients with high MAI occured within the first 5 years from the primary surgery (Fig. 2a and b).

Univariable and multivariable Cox regression analyses were performed to estimate the prognostic impact of DTC and MAI status, as well as of other clinicopathological parameters. Univariable Cox regression clearly showed that a positive DTC status prior to surgery was a significant risk factor for both reduced systemic recurrence-free survival and breast cancer–specific survial (Additional file 3: Table S1). Furthermore, persistence of DTCs after surgery also resulted in a high risk for systemic and breast cancer–specific recurrence, but the highest risk was observed for breast cancer patients with a positive DTC status both prior to and after surgery (HR = 6.93 and 8.34, respectively). MAI status was also a significant risk factor for reduced systemic recurrence-free and breast cancer–specific survival. Among other clinicopathological parameters, LN status, tumor size, grade, and age were also significant predictors of shorter survival (Additional file 3: Table S1). Multivariable Cox regression demonstrated that both pre-operative and post-operative DTC status were independent predictors of systemic recurrence-free survival and breast cancer–specific survival respectively (Table 2). Moreover, MAI score was also an independent prognostic marker of systemic recurrence-free survival and breast cancer–specific survival, with a median follow-up of 15.3 years. LN-status was, on the other hand, not a significant risk factor in the models, probably because of an association with one of the other covariates.

**Table 2:** Multivariable Cox regression of systemic recurrence-free survival and breast cancer–specific survival in operable breast cancer patients (*n* = 191) according to pre-operative DTC status after median 15.3 years of follow-up

Parameter		Hazard ratio	95% CI	*p-*value
Systemic recurrence-free survival	Pre-operative DTC status (pos. vs neg.)	2.94	1.385–6.339	**0.006**
	Post-operative DTC status (pos. vs neg.)	2.87	1.335–6.184	**0.007**
	MAI status (high vs low)	2.65	1.352–5.186	**0.005**
Breast cancer–specific survival	Pre-operative DTC status (pos. vs neg.)	3.48	1.389–8.723	**0.008**
	Post-operative DTC status (pos. vs neg.)	2.67	1.033–6.885	**0.043**
	MAI status (high vs low.)	4.35	1.916–9.874	**< 0.001**

Only results from backward stepwise selection of variables are presented. Results from overall survival are not presented.

*p*-value ≤0.05 are in bold

### Survival analysis in patients who were alive and recurrence-free after 5 years from surgery

**Fig. 3:**
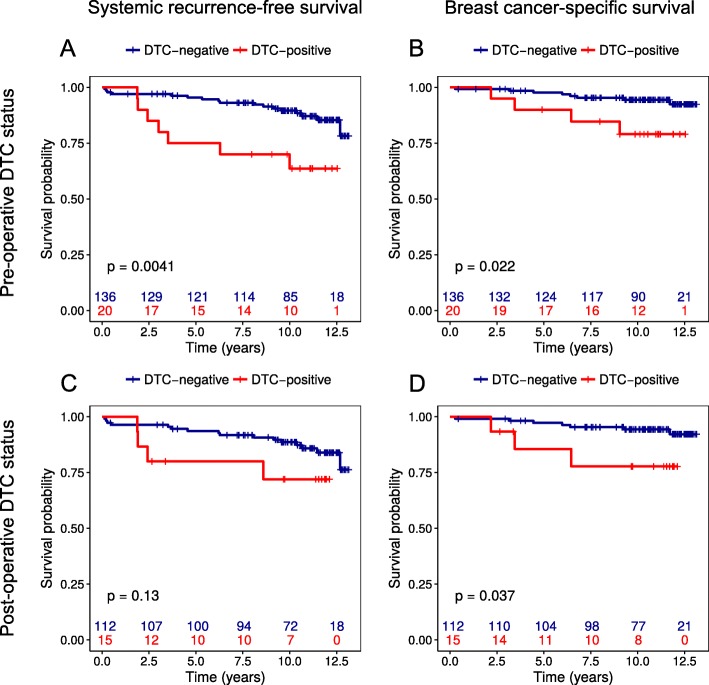
Survival analyses according to disseminated tumor cell (DTC) detection in bone marrow (BM) samples obtained prior to surgery (**a** and **b**, *n* = 156) and 3 weeks and/or 6 months after surgery (**c** and **d**, *n* = 126) from operable breast cancer patients who did not experience disease recurrence or died during the first 5 years after surgery. Median follow-up was 15.3 years, but the first 5 years were subtracted from all survival times prior to analysis

To further explore whether DTC detection can predict late disease recurrences, we excluded patients who relapsed or died within 5 years from their primary surgery and subtracted 5 years from all follow-up times from the survival analyses. A total of 156 long-term breast cancer survivors then remained in the models. Of these, 20/156 had DTCs prior to surgery, while 15/127 patients (missing data from 29 patients) had DTCs detected after surgery. Kaplan–Meier analyses showed pre-operative DTC status to be a significant predictor of late recurrences with regard to systemic recurrence-free- (*p* = 0.004) and breast cancer–specific survival (*p* = 0.022) (Fig. 3a and b). Stratification further revealed that pre-operative DTC status predicted late systemic recurrences in ER-positive patients (*p* = 0.007), pN+ patients (*p* = 0.008), patients with larger tumor size (*p* < 0.001), and patients over age 55 years (*p* = 0.027) (data not shown). For breast cancer–specific survival, pre-operative DTC status was a prognostic factor only for pN+ patients (*p* = 0.006). Post-operative DTC status, on the other hand, was a significant predictor only of reduced breast cancer–specific survival (*p* = 0.037) and not of systemic recurrence-free survival (Fig. 3c and d). Stratification also revealed that post-operative DTC status predicted late breast cancer–specific deaths in patients with pN+ disease (*p* = 0.006) and not in patients with high age or a positive ER status (data not shown). Of interest, Kaplan–Meier analyses of MAI status revealed that patients with a high MAI actually had significantly longer systemic recurrence-free survival than those with low MAI (*p* = 0.046; Fig. 2c).

Univariable Cox regression analysis confirmed pre-operative DTC status as a significant predictor of both shorter systemic recurrence-free- (HR = 3.38, *p* = 0.007) and breast cancer–specific survival (HR = 3.67, *p* = 0.034) after exclusion of early relapses and deaths. In contrast, neither post-operative DTC status nor MAI status was a significant predictor of systemic recurrence-free survival or breast cancer–specific survival. Multivariable Cox analyses further confirmed these data by showing that only pre-operative DTC status was an independent predictor of late systemic and breast cancer–specific recurrences (Table 3). High MAI, on the other hand, was independently associated with longer recurrence-free survival in this analysis restricted to late events (HR = 0.22, *p* = 0.043).

**Table 3:** Multivariable Cox regression of systemic recurrence-free survival and breast cancer-specific survival demonstrate that pre-operative DTC status is an independent prognostic factor for prediction of late recurrences (> 5 years) in operable breast cancer patients (*n* = 155). Median follow-up was 15.3 years

	Parameter	Hazard ratio	95% CI	*p*-value
Systemic recurrence-free survival	Pre-operative DTC status (pos. vs neg.)	3.27	1.314–8.149	**0.011**
	MAI status (high vs low)	0.22	0.051–0.953	**0.043**
	LN status (N1 and N2 vs N0)	2.30	0.969–5.443	0.059
Breast cancer–specific survival	Pre-operative DTC status (pos. vs neg.)	3.58	1.35–12.370	**0.044**
	LN status (N1 and N2 vs N0)	3.19	0.973–10.499	0.056

Patients with early recurrence (< 5 years) were excluded from these analyses to verify the significance of DTC detection for prediction of late disease recurrences. Only results from backward stepwise selection of variables are presented.

*p*-value ≤0.05 are in bold

## Discussion

We and others have previously shown that detection of DTCs in BM samples from operable breast cancer patients is associated with adverse clinical outcomes [14–16, 20, 26, 27]. In this study, we revisited DTC detection data from a previously described cohort, and have now collected extended long-term patient follow-up data. In addition to analysis of all data, we restricted some analyses to include only patients who were alive and recurrence-free after 5 years from surgery. A prognostic impact of DTC detection, but not MAI, could be demonstrated with regard to prediction of late disease relapse.

About 20% of clinically disease-free breast cancer patients experience disease recurrence 7–25 years after their initial diagnosis, even after mastectomy [28, 29]. In this respect, the average recurrence risk has been calculated at 4.3% per year between 5 and 12 years after postoperative adjuvant therapy [29], while the relapse risk between 10 and 20 years is about 1.5% per year [28, 29]. Although the biology behind the late recurrences in breast cancer patients is still not clearly defined, evidence indicates that BM is a common homing target for tumor cells in many types of carcinoma before they re-circulate into other distant sites [30, 31]. However, for tumor cells to survive in the BM, this microenvironment needs to be permissive for them (the metastatic niche model) [32]. To facilitate this permissiveness, the primary tumor and secondary sites seem to communicate through exosomes and direct organ tropism, modulate immune evasion, and support mesenchymal-to-epithelial transition, thus contributing to enhanced metastasis by influencing the fate of DTCs (reviewed in [33–35]). Following arrival at the BM, cellular and molecular cross-talk between the DTCs and the microenvironment further directs the DTCs into various native BM niches that also promote cell survival and dormancy [36, 37]. Recently autophagy has also been revealed as a critical mechanism for both the survival and outgrowth of the DTCs [38]. In addition to this, a large proportion of DTCs display stem cell–like features, which confer resistance to cytostatic therapy and contribute to enhanced cell survival [39]. When considering that all these factors contribute to survival of latent/dormant DTCs it reveals the molecular complexity of breast cancer, which further challenges both the choice and the success of adjuvant treatment for long-term survival.

To our knowledge, our study has the longest median follow-up reported in an analysis of DTCs in breast cancer. Only one other study has analysed the prognostic value of DTC status with regard to relatively long follow-up. In that study, 189/350 (54%) patients, of whom 31% were DTC-positive, relapsed during a median 12.5 years of follow-up [17]. This is comparable to 33% of the DTC-positive patients relapsing in our study. However, in their study, DTC detection was a significant independent prognostic factor for prediction of early relapses occurring 0–5 years from surgery and not for late relapses (> 5 years from surgery) [17], in contrast to our findings.

We show that the presence of DTCs in BM before surgery is a significant predictor of late recurrences and thus reduced systemic recurrence-free and breast cancer–specific survival in operable breast cancer patients. Stratification further demonstrated that pre-operative DTC status was particularly predictive of reduced survival in postmenopausal women (*p* = 0.027), patients with ER-positive disease (*p* = 0.007), lymph node involvement (*p* = 0.008), and large tumors (*p* < 0.001) by the log-rank test. Hence, our results support the fact that ER-positive patients are at particular risk of experiencing late recurrences, and extended endocrine therapy from 5 to 10 years is now recommended for this patient group [40]. A recent report from the International Breast Cancer Study Group, in which the hazard rates of breast cancer recurrence were estimated from 4105 breast cancer patients and 24 years of follow-up, also showed that the hazard for experiencing late relapse remains elevated and fairly stable beyond 10 years in ER-positive patients [41]. Other studies also support this conclusion (reviewed in [36, 42, 43]). However, because the ER-positive patient group is heterogeneous, differences have further been demonstrated between pre- and post-menopausal women based on molecular characteristics. In this respect, it has been specified that most cases of late recurrences arise in postmenopausal ER-positive women aged 60 years or older [42, 44]. Nevertheless, our study did not confirm ER-status as an independent prognostic factor, in contrast to pre-operative DTC status.

Previously, we and others have shown that both MAI scoring and DTC detection give independent prognostic information in operable breast cancer patients [24]. Because proliferation is a key driver for cancer progression, and substantial variability in Ki67 scoring is well known to occur [45], we wanted to extend our study to include investigations of MAI as a marker for prediction of late recurrences. High proliferation is associated with a more aggressive disease, so one prediction would be a higher relapse rate among these patients, as well as more frequent systemic disease and thus a positive DTC status. Our data support a significant association between high MAI score and relapse (Fig. 3) but no significant association between high MAI and DTC positivity (data not shown). This finding is probably because patients with high proliferation in the primary tumor largely seem to experience early disease relapse, within 5 years from diagnosis, in contrast to DTC-positive patients, who experience both early and late relapses [13]. Proliferation as a marker for prediction of early relapse has also been shown in other studies [46, 47]. Moreover, several molecular multi-gene assays including proliferation markers, among other markers (such as Mammaprint [48], Oncotype Dx Recurrence Score [49], Genomic Grade Index [50], Prosigna PAM50 Risk of Recurrence Score [51], Breast Cancer Index [52] and EndoPredict [53]) also support this. These assays were developed originally to give an overall risk assessment of recurrence by providing prognostic information not contained in the clinicopathological parameters. However, with a few exceptions, these multi-gene assays provide prognostic information restricted only to the first 5 years after the diagnosis (reviewed in [54]). This situation illustrates the challenges of accurate classification of primary breast tumors for prediction of late recurrences and suggests that DTC assessment may supplement primary tumor diagnostics in prognostic stratification. A few studies comparing the risk assessment by multi-gene assays and presence of DTCs in the bone marrow of operable breast cancer patients also support this as they did not find any association between them [55, 56]. On the contrary, another study did show that DTC detection was associated with a high Oncotype DX recurrence score [57]. Further studies are warranted.

Characterisation of DTCs and CTCs has revealed a challenge in current adjuvant treatment: the choice of targeted/adjuvant therapy in almost every solid cancer is largely based on an initial tissue biopsy obtained from the primary tumor. However, primary tumor characteristics do not necessarily reflect the characteristics of the metastasising DTCs and CTCs due to tumor cell heterogeneity and acquired evolutionary changes in the DTCs/CTCs during treatment. This has been demonstrated in several breast cancer studies, especially with regard to HER2 and ER status [58–61]. HER2-positive DTCs/CTCs have been detected in patients with an apparently HER2-negative primary tumor, resulting in patients who in fact are eligible for HER2-based treatment [62, 63]. The same has been shown for ER status [60, 61, 64, 65]. DTCs and CTCs may be ER-negative and PR-negative despite originating from a hormone receptor–positive tumor, possibly explaining the failure of endocrine therapy in a subset of ER-positive patients and vice versa. To overcome the issue of tumor cell heterogeneity, it has in the recent years been much focus on detection of circulating cell-free tumor DNA (ctDNA) from plasma of cancer patients. ctDNA is extracellular DNA that may originate from apoptotic and necrotic tumor cells in the primary tumor, metastatic lesions, or CTCs/DTCs in the circulation. In this respect, the ctDNA pool should be representative of the total tumor burden in an individual cancer patient, and several studies have shown both a prognostic and a predictive value of ctDNA detection in breast cancer patients (e.g. [66, 67]). Because ctDNA analysis is easily performed, without the need for enrichment and isolation of rare cancer cells, it is likely to be the preferred option for genotyping and monitoring of treatment response in the future. Further investigations are, however, needed to elucidate whether ctDNA assessment can predict late recurrences of breast cancer similarly to or better than DTC detection. Nevertheless, analyses of CTCs and DTCs provide a unique opportunity for in-depth assessment of viable metastasising tumor cells and their interaction with the tumor microenvironment, providing access to information that cannot be revealed using ctDNA.

## Conclusion

Our data show that the presence of DTCs prior to surgery is an independent predictor of late disease recurrences and thus reduced systemic recurrence-free- and breast cancer–specific survival after a median 15.3 years of follow-up. In contrast, persistence of DTCs after surgery is a significant predictor only of late breast cancer recurrences. Proliferation, on the other hand, seems best to predict early relapse. Because the ultimate goal of all clinical research is to improve patient outcomes, further molecular characterisation of the DTCs and microenvironmental factors influencing on survival of dormant DTCs as well as dormancy exit mechanisms is required to better identify breast cancer patients at high risk of developing late disease relapse and thus in need of extended therapy.

## Supplementary information

**Additional file 1 Figure S1**: Kaplan–Meier overall survival estimates according to presence of disseminated tumour cells (DTCs) in bone marrow (BM) samples from operable breast cancer patients before as well as after (3 weeks and/or 6 months) surgery. A) and B) DTC status in all patients (*n* = 191), median 15.3 years follow-up. C) and D) DTC status in patients (*n* = 156) who did not experience disease recurrence or died during the first 5 years after surgery, and where the first 5 years were subtracted from all survival times prior to analysis.

**Additional file 2 Figure S2**: Overall survival estimates according to high (MAI ≥ 10) or low (MAI < 10) proliferation measured by the mitotic activity index (MAI) in tissue from operable breast cancer patients. A) MAI status in all patients (*n* = 191), median 15.3 years of follow-up. B) MAI status in patients (*n* = 156) who did not relapse or die during the first 5 years after surgery, and where the first 5 years were subtracted from all survival times prior to analysis.

**Additional file 3 Table S1**: Risk factors for reduced systemic recurrence-free- and breast cancer–specific survival in operable breast cancer patients (*n* = 191) with a median 15.3 years of follow-up revealed by univariable Cox regression.

## Data Availability

The underlying datasets will be made available upon request to Dr. Kjersti Tjensvoll after approval from the regional ethical committee.

## Abbreviations

BM: Bone marrow
CTC: Circulating tumor cells
DTC: Disseminated tumor cells
ER: estrogen receptor
HR: Hazard ratio
MAI: Mitotic acitivity index
