# Supplementary material for: Detection of disseminated tumor cells in bone marrow predict late recurrences in operable breast cancer patients
Source: BMC Cancer. 2019 Nov 21;19:1131. doi: 10.1186/s12885-019-6268-y (PMC6873493; doi:10.1186/s12885-019-6268-y)
Supplement: Supplementary file 3 — Additional file 3 Table S1: Risk factors for reduced systemic recurrence-free- and breast cancer–specific survival in operable breast cancer patients (n = 191) with a median 15.3 years of follow-up revealed by univariable Cox regression. [file 12885_2019_6268_MOESM3_ESM.docx]

**Table S1:** Risk factors for reduced systemic recurrence-free- and breast cancer–specific survival in operable breast cancer patients (*n* = 191) with a median 15.3 years of follow-up revealed by univariable Cox regression.

| **Parameter** | **Hazard ratio** | **95% CI** | ***p-*value** |
| --- | --- | --- | --- |
| ***Systemic recurrence-free survival*** |  |  |  |
| Pre-operative DTC status (pos vs neg) | 3.48 | 1.90***–***6.34 | ***<0.001*** |
| Post-operative DTC status (pos vs neg) | 2.96 | 1.47***–***5.96 | ***0.002*** |
| Pre- and post-operative DTC status (pos vs neg) | 6.93 | 3.06***–***15.70 | ***<0.001*** |
| MAI status (high vs low) | 1.96 | 1.11***–***3.48 | ***0.021*** |
| Age (>55 years vs <55) | 1.14 | 0.50***–***1.54 | 0.648 |
| Tumour size (T4 and T3 vs T2 vs T1) | 2.97 | 1.15***–***7.65 | ***0.024*** |
| LN status (N2 and N1 vs N0) | 2.85 | 1.62***–***5.02 | ***<0.001*** |
| Grade (3 vs 2 vs 1) | 1.87 | 0.89***–***4.02 | 0.100 |
| ER status (pos vs neg) | 1.60 | 0.09***–***4.57 | 0.643 |
| PR status (pos vs neg) | 2.26 | 0.31***–***16.74 | 0.424 |
|  |  |  |  |
| ***Breast cancer–specific survival*** |  |  |  |
| Pre-operative DTC status (pos vs neg) | 3.70 | 1.86***–***7.36 | ***<0.001*** |
| Post-operative DTC status (pos vs neg) | 3.26 | 1.47***–***7.22 | ***0.004*** |
| Pre- and post-operative DTC status (pos vs neg) | 8.34 | 3.59***–***19.35 | ***<0.001*** |
| MAI status (high vs low) | 3.45 | 1.78***–***6.80 | ***<0.001*** |
| Age (>55 years vs <55) | 1.12 | 0.46***–***1.74 | 0.739 |
| Tumour size (T4 and T3 vs T2 vs T1) | 1.71 | 1.04***–***2.83 | ***0.035*** |
| LN status (N2 and N1 vs N0) | 4.02 | 2.03***–***7.96 | ***<0.001*** |
| Grade (3 vs 2 vs 1) | 1.90 | 1.22***–***2.92 | ***0.004*** |
| ER status (pos vs neg) | 2.41 | 1.18***–***4.95 | ***0.016*** |
| PR status (pos vs neg) | 1.19 | 0.61***–***2.34 | 0.607 |
